# Acoustic and Ultrasonic Sensing Technology in Non-Destructive Testing

**DOI:** 10.3390/s25113271

**Published:** 2025-05-22

**Authors:** Sergio Castiñeira-Ibáñez, Daniel Tarrazó-Serrano, Constanza Rubio

**Affiliations:** Centro de Tecnologías Físicas, Universitat Politècnica de València, Camí de Vera s/n, 46022 València, Spain; sercasib@fis.upv.es (S.C.-I.); crubiom@fis.upv.es (C.R.)

## 1. Introduction

The physics of acoustics, particularly the high-frequency domain of ultrasonics, remains a dynamic area of innovation in sensing technologies. Ultrasonic waves are mechanical oscillations propagating through matter at frequencies beyond the range of human hearing. They offer a unique ability to interrogate the interior of materials and structures non-invasively. Because they are sensitive to mechanical, structural, and elastic properties, ultrasonic waves are an ideal tool for applications that require internal inspection without direct access or physical intrusion. This makes them especially valuable in the context of non-destructive testing (NDT), structural health monitoring, and, increasingly, biological and environmental sensing. Ultrasonic wave propagation is governed by classical elastic wave theory, where energy can travel through solids in various modes such as longitudinal, shear, surface, or guided wave modes. Guided wave modes, such as Lamb waves, are particularly well suited for plate-like or multilayered structures, as they can propagate over long distances while maintaining high sensitivity to geometric or material discontinuities. These characteristics have made guided waves a cornerstone of modern NDT approaches, especially in large infrastructure systems such as storage tanks, pipelines, and railway tracks [1,2]. Among the most established ultrasonic techniques, acoustic emission (AE) is lauded for its ability to detect transient elastic waves generated by the rapid release of energy in materials under stress. Unlike conventional ultrasonic testing methods that rely on externally introduced signals, AE leverages the material’s own response to structural changes such as crack initiation, fiber breakage, or corrosion. This passive approach enables the continuous, real-time monitoring of critical components and has been widely adopted in applications ranging from pressure vessels and pipelines to aerospace structures [3]. AE complements guided wave and imaging techniques, particularly in damage localization and early failure detection scenarios [4].

Numerous contributions to this Special Issue reflect the central role of ultrasonic-guided waves in contemporary sensing applications. Advances include their use in detecting damage in the bottom plates of storage tanks, assessing internal stiffness in fiber-reinforced composites, identifying flaws in multilayer structures, and monitoring defects in railway systems. The versatility of guided waves, particularly when combined with advanced signal processing and sensor arrays, enables the efficient inspection of difficult-to-reach areas with minimal intervention [5]. Parallel to improvements in wave propagation techniques, significant sensor and transducer design developments continue to shape the field. Piezoelectric ceramics such as PZT remain widely used, but new materials, such as flexible polymers and polymer composites, are gaining ground due to their adaptability and mechanical robustness. Some articles in this Special Issue explore novel damping geometries, surface acoustic wave devices, and optimized array configurations. This shows how hardware design remains crucial to improving signal-to-noise ratios and imaging resolution [6].

Beyond classical inspection, integrating computational intelligence into ultrasonic systems is redefining the research landscape. Several studies in this Special Issue apply deep learning architectures, such as convolutional neural networks, to ultrasonic signal interpretation. These data-driven methods offer superior performance in identifying and quantifying defects, particularly in noisy environments or when handling complex, high-dimensional data [7]. For example, recent research applies neural networks to extract microcrack information from wavefield interactions [8] or to perform quantitative crack sizing in pipelines based on one-dimensional time-series data [9].

Advances in materials science, particularly in additive manufacturing, have introduced complex and often anisotropic structures that demand tailored acoustic evaluation strategies. At the same time, ultrasonic methods are expanding beyond traditional solid media to include aquatic and volumetric environments, requiring new imaging and reconstruction techniques suited for submerged or irregular geometries [10]. NDT is also relevant in environmental monitoring, where acoustic measurements can capture structural changes in soils or geological formations and provide early indicators of instability or failure. Moreover, the high sensitivity of new acoustic systems to minute vibrations and emissions has enabled novel applications in biological and ecological contexts, such as detecting activity in insects, plants, or small animals. Recent advances include configurable ultrasonic lenses designed for subwavelength resolution and beam shaping, which leverage geometric design and material properties to control wave propagation with high precision [11]. Techniques for tunable beam bending and directional focusing, such as those using Janus-type acoustic structures, have been explored to enable flexible energy steering in complex scenarios [12]. These innovations are beneficial for scanning hidden regions or navigating obstacles and are applicable in both industrial and medical contexts. Among the emerging concepts in subwavelength focusing, photonic nanojets represent a promising direction for future ultrasonic imaging enhancement. Generated by dielectric microspheres under optical illumination, these highly localized and high-intensity beams can be coupled with acoustic systems to improve spatial resolution and sensing accuracy. While not yet featured in the contributions of this Special Issue, their potential integration into optoacoustic or hybrid systems is likely to influence next-generation sensor designs [13].

Altogether, the articles presented in this Special Issue reflect a growing trend toward integrating ultrasonic technologies with innovative sensing strategies, data-driven processing, and interdisciplinary applications. Whether used for inspecting the integrity of critical infrastructure, analyzing manufactured components, or monitoring complex biological and geological systems, ultrasonic sensors are becoming more adaptive, accurate, and multifunctional. The convergence of classical acoustic physics with artificial intelligence, flexible materials, and advanced imaging techniques ensures that acoustic and ultrasonic sensing technologies will remain at the forefront of scientific and technological development.

## 2. An Overview of Published Articles

This Special Issue brings together seventeen original contributions that reflect the state-of-the-art in acoustic and ultrasonic sensing for NDT. Throughout this section, the numbers in parentheses (e.g., (1), (2), etc.) refer to the individual articles included in this issue, as listed in Section “List of Contributions”. The research spans sensor design, advanced signal processing, machine learning integration, and novel applications across civil, mechanical, and biological domains. For clarity, we have organized the papers into thematic categories and summarize key results.

### 2.1. Structural Health Monitoring and Damage Detection

Several papers focus on damage identification using guided waves and acoustic emission (AE). Xu et al. (1) applied the Lamb Wave Mode of AE to monitor impact damage in epoxy glass fiber plates, showing that damage-induced AE can be directly extracted from impact signals in real time. Shen et al. (4) integrated guided wave detection with a one-dimensional convolutional neural network (CNN) to detect pipeline cracks, achieving errors below 2% in both the simulation and experiment.

Adams et al. (10) used guided waves to assess the stiffness of fiber-reinforced composites non-destructively. Their inverse numerical model estimated matrix components with less than 10.4% error and tensile/flexural stiffness with average errors of 3.6% and 9.0%, respectively. Ma et al. (17) introduced a collaborative method using sensor arrays on a tank bottom and wall plates to locate corrosion-induced defects in storage tanks. A correlation-based signal processing approach improved image clarity and achieved localization errors as low as 5.4%.

In rail systems, Zeng et al. (9) developed a chaotic oscillator model based on the Duffing system to identify ultrasonic guided wave signals. Notches of 0.46 mm and 1.78 mm were detected at the rail base and head, respectively, with Kolmogorov entropy used as a quantitative index. Tumšys et al. (14) investigated delamination in multilayer composites using the asymmetric Lamb wave $A_{0}$ mode, demonstrating its sensitivity to layer separation.

### 2.2. Sensor and Transducer Development

Sensor design is a recurring theme. Vechera et al. (2) studied the effect of snubber geometry in piezoelectric ultrasonic transducers (PETs). A truncated cone-shaped damper increased the transmitter’s bandwidth and transient performance by modifying the inclination angle α of the generatrix. Xiong and Qi (3) proposed a grating interferometric acoustic sensor based on a flexible polymer diaphragm. It achieved a minimum detectable pressure of 164.8 $\mu Pa/\sqrt{Hz}$ and a flat frequency response with less than 3.2% jitter in the speech range, suggesting applications in acoustic monitoring and voice acquisition.

Brand and Drese (5) introduced a laser-induced optoacoustic system using a photorefractive interferometer. Phase velocities and attenuations of longitudinal waves were measured across a 3–55 MHz range with relative errors under 0.2% for materials, such as silicon and aluminum. Schulmeyer et al. (7) developed a dual-mode surface acoustic wave (SAW) delay line on a 64° Y-cut lithium niobate substrate, capable of differentiating between liquid water and ice while simultaneously measuring temperature, proving effective in harsh environments.

### 2.3. Signal Processing and Machine Learning

Several articles showcase the power of data-driven analysis. Shen et al. (4) leveraged CNNs for crack detection, while Dolmatov and Zhvyrblya (6) optimized the design of sparse matrix phased arrays for the total focusing method (TFM). Their approach reduced the data volume by up to 84% without sacrificing image quality.

Malashin et al. (11) proposed a hybrid experimental–computational method for evaluating acoustic anisotropy in additively manufactured materials. A new anisotropy coefficient, derived from transverse wave velocities, showed a strong correlation (0.97) with echo amplitude variations. A neural network optimized via genetic algorithms efficiently predicted this coefficient. Moreh et al. (16) designed a deep learning model for detecting microcracks from wavefield data. Their asymmetric encoder–decoder network, enhanced with feature reuse and manifold visualization, reached a detection accuracy of 87.74%.

### 2.4. Cross-Disciplinary and Emerging Applications

This issue also highlights innovative uses of ultrasonic sensing. Huang et al. (8) performed a bibliometric review of ultrasonic In-Line Inspection (ILI) for oil and gas pipelines. The paper visualizes research trends and classifies defect detection techniques, offering a comparative framework from lab testing to industrial deployment.

Luo et al. (12) presented a sonar-based 3D reconstruction system for submerged bridge abutments. Using MS1000 sonar and automated processing, they extracted coordinates of contours and reconstructed abutments with volume estimation errors of 13.56% (holes) and 10.65% (spalling). Zhu et al. (13) simulated landslides in the lab to analyze AE and micro-seismic signal patterns. They proposed a classification method to separate burst and continuous AE signals, revealing their evolution as a precursor to failure.

Finally, Turov et al. (15) used distributed acoustic sensors (DASs) to monitor the sounds of the Madagascar hissing cockroach. Both the insect hissing and mechanical interactions with optical fibers were recorded, which could lead to potential new innovations in agricultural or ecological monitoring using fiber-optic acoustic sensing.

## 3. Conclusions

This Special Issue, devoted to acoustic and ultrasonic sensing technology in non-destructive testing, presents a comprehensive and up-to-date overview of state-of-the-art research in the relevant field. The seventeen contributions not only demonstrate significant progress in conventional techniques, but also incorporate emerging approaches such as signal processing through artificial intelligence, innovative sensor and transducer design, and interdisciplinary applications. The topics covered in this collection clearly align with the thematic areas outlined in the section, “An Overview of Published Articles”, including structural health monitoring and damage detection, sensor and transducer development, advanced signal processing, and machine learning, as well as novel applications across civil, mechanical, biological, and environmental domains. This thematic diversity underscores the ongoing evolution of ultrasonic sensing toward more adaptive, accurate, and multifunctional solutions, reaffirming its central role in current scientific and technological innovation. We believe that these seventeen contributions add great value to the scientific advancement of non-destructive testing. We thank all authors for the meticulous reviewing of their research.
